# Understanding perinatal patient’s health preferences and patient-provider relationships to prevent congenital syphilis in California and Louisiana

**DOI:** 10.1186/s12884-022-04883-w

**Published:** 2022-07-11

**Authors:** Jennifer A. Wagman, Eunhee Park, Gloria P. Giarratano, Pierre M. Buekens, Emily W. Harville

**Affiliations:** 1 Fielding School of Public Health, Department of Community Health Sciences, University of California, 650 Charles E Young Dr S, Los Angeles, CA 90095 USA; 2 Louisiana State University Health Sciences Center, School of Nursing, 1900 Gravier St, New Orleans, LA 70112 USA; 3 School of Public Health and Tropical Medicine, Department of Epidemiology, Tulane University, 1440 Canal Street, Suite 2000, New Orleans, LA 70112 USA

**Keywords:** Syphilis, Congenital, Prenatal care, Pregnancy, Sexually transmitted infection, Qualitative research, Health disparities, Health preferences

## Abstract

**Background:**

Congenital syphilis (CS) has reemerged as a global maternal and child health crisis. Kern County, California and East Baton Rouge Parish, Louisiana are among the highest CS morbidity regions in the United States. We previously reported on social-ecological and structural barriers to prenatal care and maternal syphilis testing and treatment in these two regions. The aim of this study was to examine perinatal patient’s health preferences and perceptions of patient-provider relationships in the prenatal care clinic setting.

**Methods:**

Between May 2018 and January 2019 we conducted 20 in-depth qualitative interviews with prenatal providers and 8 focus group discussions with pregnant and postpartum individuals in Kern County and East Baton Rouge Parish. We applied an adapted health services framework to analyze participants’ understanding of health disparities and vulnerable populations; perinatal patient’s health and prenatal care preferences; and participants’ perspectives of clinical encounters in the context of prenatal care and maternal syphilis testing and treatment.

**Results:**

Site-specific determinants of syphilis infection emerged but participants from both locations felt CS prevention efforts should be prioritized among youth, racial/ethnic minority populations, people experiencing socioeconomic limitations and people with other commonly occurring health conditions. Although perinatal patients expressed clear health preferences, they reported inconsistent receipt of respectful, patient-centered care. Inconsistencies were connected with limited ethnic and cultural competence among providers, and implicit, negative attitudes toward patients using substances, experiencing homelessness, or engaging in sex work. Providers clearly aimed to offer high quality prenatal care. However, some clinic and health systems level factors were thought to reduce positive and communicative patient-provider relationships, contributing to gaps in use of prenatal care and syphilis testing and treatment.

**Conclusions:**

Our findings suggest that interventions tailored to address setting-specific determinants (including clinic and health system factors) of disparities in CS risk could improve pregnant people’s access to prenatal care and ensure they and their sex partners receive timely syphilis screening and treatment. We recommend all prenatal care providers receive training on how to identify and mitigate implicit biases and provide competent and compassionate patient-centered care.

## Background

Syphilis is a sexually transmitted infection (STI) caused by the bacterium *Treponema pallidum,* which can be transmitted during vaginal, anal, or oral sex, and transplacentally to a fetus during pregnancy (i.e., congenital syphilis). Congenital transmission can occur at any point in gestation and any stage of maternal syphilis infection, but the risk is highest in pregnant people with primary or secondary syphilis. Congenital syphilis (CS) is a critical, yet under-addressed public health concern in the United States (U.S.) and globally as it is associated with extensive and significant morbidity and mortality. If untreated or inadequately treated, CS can lead to miscarriage, stillbirth, prematurity, low birth weight, or death shortly after birth. Babies born with CS who survive are at increased risk for deformed bones, severe anemia, enlarged liver and spleen, jaundice, brain and nerve problems, meningitis, and skin rashes [1, 2].

Evidence-based prevention, diagnostic and treatment guidelines for STIs, including CS, are available and regularly updated [1, 3]. CS can be prevented through timely identification and treatment of a pregnant person with syphilis, using a regimen of Benzathine penicillin G. Nonetheless, between 2013 and 2019 in the U.S., the number of CS cases increased by 417% (from 362 to 1870 cases) and the rate of CS increased from 8.7 to 48.5 cases per 100,000 births. Also during this period, a parallel rise occurred in the rate of primary and secondary syphilis infection among people who identified as women [4].

In 2017, the U.S. Centers for Disease Control and Prevention (CDC) released a “call to action” to address the nation’s rising rates of syphilis and CS infection [5], at which time, California was the state with the highest number of CS cases (281) and Louisiana had the highest incidence rate of CS infections (93.4 cases per 100,000 live births) [3]. The CDC’s call outlined specific actions for researchers and public health practitioners to develop and improve approaches for reducing syphilis and addressing barriers to vulnerable pregnant people’s early and adequate use of prenatal care [5]. In response, researchers from University of California (UC), California State University (CSU), Tulane University and Louisiana State University Health Sciences (LSUHSC) launched a two-site study in 2018 to examine knowledge, attitudes and behaviors related to CS and its prevention in California and Louisiana. Through 2019, we conducted qualitative research with prenatal care providers (PCP) and pregnant and postpartum (i.e., perinatal) participants in two of the highest CS morbidity regions in California (Kern County) and Louisiana (East Baton Rouge Parish).

We previously published setting-specific findings on providers’ and perinatal participant’s perspectives on congenital syphilis as well as social-ecological and structural barriers to prenatal care and maternal syphilis testing and treatment in Kern County [6] and Baton Rouge [7]. Consistent with evidence from other U.S. locations [8, 9], we found an interplay of individual (e.g., socioeconomic status or SES), relationship (e.g., partner notification), community (e.g., sexual health education), and societal/structural factors (e.g., immigration policies and public health program funding) that either put people at risk for or protected them from acquiring or transmitting syphilis [6, 7]. At the core of these determinants were a range of disparities, including racial and ethnic differences in risk for infection and access to prenatal care and treatment for syphilis - differences that were mirrored across the U.S. When we launched our study in 2018, national rates of CS incidence were highest in pregnant people who identified as Black (87 cases per 100,000 live births), American Indian/Alaskan Native (79 cases per 100,000 live births), and Hispanic/Latinx (45 cases per 100,000 live births) [9].

Health disparities are defined by the National Institute on Minority Health and Health Disparities as health differences, on the basis of one or more health outcomes, that adversely affect disadvantaged populations [10]. Understanding and responding to health disparities - such as the racial and ethnic differences observed in CS risk - is fundamental to the success of any intervention designed to reduce or end the epidemic. Additionally, it is necessary to recognize and intervene on *other* disparities in CS risk, such as those at the health care system level. For instance, studies have found that quality of prenatal care and maternal health outcomes are adversely impacted by patient and provider-level factors, including poor communication between pregnant people and their prenatal provider [11], and limited provision of patient-centered care (i.e., care that is respectful of and responsive to patient preferences, needs, and values [12]. However, very little research has examined the relationship between disparities in CS risk and pregnant patients’ health preferences or assessed clinical interactions and provision of patient-centered care in the context of CS prevention and treatment during prenatal care.

In this paper we present qualitative findings from in-depth interviews conducted with prenatal care providers and focus group discussions conducted with perinatal patients in Kern County and East Baton Rouge. We describe their perspectives on and experiences of patient-centered care and the patient-clinician relationship in the context of syphilis prevention and treatment during prenatal care in both settings. We used a health services analytic framework that was developed to reduce disparities in the context of the health care system [13] to assess how participants defined ‘health disparities’ and ‘vulnerable populations’ and explore perinatal patient’s preferences for health and prenatal care - referring to their health choices that reflected ‘the relative desirability of a range of health experiences, treatment options and health states’ [14] and perinatal patient’s and providers’ feelings about patient-provider clinical encounters. Toward the goals of health services research to organize, manage, and deliver high-quality care [15], we provide evidence-based recommendations. Although our findings are specific to Kern County and East Baton Rouge Parish, we believe they can be translated to many other settings in the U.S. and help researchers and practitioners develop and implement interventions within and in collaboration with health care systems, to prevent disparities in risk for CS and access to high quality prenatal care and STI testing and treatment.

## Methods

### Research setting

This was a two-site study, conducted between May 2018 and January 2019 in Kern County, California and East Baton Rouge Parish, Louisiana.

Despite representing only 2% of California’s 2018 population of 893,758 people [16], 17% of all CS cases in the state were in Kern County [17], a mostly rural, agricultural region. More than half of the population identified as Hispanic/Latinx (56%), followed by Non-Hispanic/Latinx White (33%), Black/African American (5%), Asian (5%) and Multiracial people [18]. Among 329 people who had babies with CS in California in 2018, the highest case prevalence (155 or 47%) was among Hispanic/Latinx persons, but the highest incidence rate (286 per 100,000 live births) was among Black/African American persons [17].

In 2018, the Baton Rouge region of Louisiana had the third highest prevalence of CS cases in the state and within Baton Rouge, the highest prevalence of primary and secondary syphilis was in the East Baton Rouge Parish [19]. The Parish’s estimated 2018 population was 442,058 people [20] and, distinct from Kern, it is mostly urban with only 4% of the population identifying as Hispanic/Latinx [21]. The largest racial/ethnic groups are Non-Hispanic/Latinx Blacks/African Americans (47%) and Whites (43.8%) [21]. Among 43 people who had CS babies in Louisiana in 2018, the highest case prevalence (33 or 77)% as well as the highest incidence rate (150 per 100,000 live births)^1^ was among Black/African American persons [22].

### Research design

A multiple principal investigator approach was used, allowing for shared leadership by UC San Diego School of Medicine and Tulane University School of Public Health and Tropical Medicine (Tulane). All research instruments and activities were developed and implemented with input from local health departments, and in partnership with researchers from CSU Bakersfield and LSUHSC. The project was part of a cooperative agreement between the March of Dimes and CDC. All research protocols and instruments were approved by Institutional Review Boards at UC San Diego and Tulane and final clearance was provided by the CDC’s Office of Management and Budget.

This was a qualitative study and two research methods were used at each site: one-to-one in-depth interviews (IDI) with PCPs serving high-risk pregnant patients and focus group discussions (FGD) with high-risk perinatal patients. We used the CDC’s definition of high-risk to include people with a history of syphilis infection, incarceration, drug use, or multiple or concurrent partners, and those in areas with high rates of syphilis [23].

#### In-depth interviews with prenatal care providers

We conducted IDIs with prenatal care providers to gather individual-level information about their experiences surrounding testing and treating syphilis in the perinatal care setting. Meeting one-on-one allowed us to understand each PCP’s level of knowledge, personal attitudes and professional experiences and enabled us to compare and contrast findings by setting and type of PCP. We considered conducting FGDs with PCPs but it was not feasible given their busy and inflexible clinic schedules. Further, in Kern County these providers were geographically spread apart and it was hard to find a common location and day and time where all PCPs could meet.

California IDI participants were recruited from Kern County Public Health Department, Omni Family Health, Kern Medical Center, and Clinica Sierra Vista Health Care Services by sending direct emails or telephoning each site’s medical director. In Louisiana, participants were recruited from Woman’s Hospital (which provides prenatal, intrapartum, and postnatal care services) and Baton Rouge Community Health Center, by introducing the study at a staff meeting at Woman’s Hospital and directly contacting community clinics. Minimum eligibility criteria were: (1) PCP in Kern County or East Baton Rouge Parish in current position for more than 6 months; (2) Currently working directly (at least 50% of the time) with high-risk pregnant people; (3) Having a phone or another way of being contacted.

IDIs were conducted in English, in private, with one research participant at a time who was interviewed by a member of the research team. All participants provided written consent. A semi-structured guide was used to focus each discussion on the provider’s clinical training for and knowledge of syphilis and its management during pregnancy; the protocol(s) they used for STI testing and treatment, and their opinions of patients’ health-seeking behavior. IDIs were audio recorded with participant consent. IDIs lasted approximately 60 minutes and providers were compensated with a $50 gift card.

#### Focus group discussions with pregnant and postpartum participants

We chose to conduct focus groups with pregnant and postpartum people to gather group-level information on perceptions, insights, attitudes, and experiences surrounding sexual and reproductive health, prenatal care preferences, and perinatal clinical encounters. Although individual interviews are typically preferred for researching sensitive and personal topics, such as sexual health, our health department colleagues shared that knowledge about syphilis and other STIs was low in the local population. As a result, we felt FGDs would be ideal for fostering thought and conversation among participants about topics they had not previously considered, in depth. Specifically, our approach was to bring participants together and provide space for bouncing ideas off each other and having more in-depth discourse than they might have done on their own in an individual interview.

In Kern County, FGD participants were recruited from the same health clinics/centers from where providers were drawn, a residential perinatal center providing substance use recovery services, a transitional sober living community, and an intimate partner violence/domestic violence center. At each site, we provided the clinic manager/director details on the study and left information sheets containing descriptions of the study and contact details. Staff were asked to share details of the study with their clients/residents. Additional participants were recruited through snowball sampling, a nonrandom technique where initial participants identify additional study participants.

In East Baton Rouge, FGD participants were recruited from Family Road of Greater Baton Rouge, an agency that provides services to families, including coordinating enrollment in Medicaid, Women’s, Infants, and Children nutritional program (WIC), Supplemental Nutrition Assistance Program (SNAP), and Healthy Start. Family Road caseworkers informed clients of the FGDs and posted recruitment flyers. Staff and providers at a residents’ clinic at Woman’s Hospital promoted study recruitment to patients.

Minimum eligibility criteria for FGD participants included: (1) Aged 18 years and older; (2) Currently pregnant or delivered an infant less than 12 months prior; (3) receiving prenatal or early postpartum care in Kern County or East Baton Rouge Parish; (4) Having been a resident of Kern County or East Baton Rouge Parish for at least 6 months; (5) Having a phone or another way of being contacted.

All FGDs were conducted in person, in a private location with a group of participants who were led through discussion by a moderator and a note-taker/assistant from the research team, both of whom were bilingual in English and Spanish. All participants provided written consent. A semi-structured guide was developed in English and translated into Spanish and used to focus discussion on participant’s knowledge of STIs; health information-seeking behaviors; awareness of the syphilis and CS epidemics; forms of patient-provider communication; experiences accessing prenatal care; health beliefs and preferences; health insurance coverage; and perceptions of clinical encounters. One FGD in Kern County was conducted in Spanish and all others were conducted in English. Per recommendations for developing research materials for non-English speaking participants [24], the consent form and guide for the FGD conducted in Spanish were developed with the assistance of a research team member with native and advanced proficiency in both English and Spanish, who also had familiarity with the goals for the ethical conduct of research and the principles and concepts of informed consent. To increase comprehensibility and clarity, we followed recommendations for dual language research guides [24] by writing the consent form and FGD guide in active voice, using simple sentence structures, and avoiding complex and technical language. Further, we back translated (from Spanish to English) both instruments and carefully compared them to the original English materials. Because we anticipated that many FGD participants used both languages interchangeably, we maintained key terms in both English (e.g., sexually transmitted infection) and Spanish (e.g. infección transmitida sexualmente).

All FGDs were audio recorded with participants’ consent. On average, there were 10 participants per group (the number of participants per FGDs ranged from 5 to 15 people) and FGDs took 60–90 minutes. In compensation for their time, pregnant and postpartum participants received a $25 gift card.

#### Demographic information

We collected demographic data from all participants after they signed the consent form and before starting the interview or focus group. PCPs provided data on type of practice they worked in, the number of years in their current practice, the race /ethnicity / income level of most of their patients, and percent of their patients who were uninsured or publicly insured. Perinatal participants provided their age, pregnancy status, race/ethnicity, and annual income. We organized income data using the family income format from the Current Population Survey (CPS) administered by the U.S. Census Bureau [25]. PCPs reports of their patients’ income was organized in three categories (under $20,000, between $20,000 and $49,999 and $50,000 or higher). Perinatal participants’ income data was gathered using an adapted version of the categories from the 2019 CPS Income and Poverty Report [26], limited to six categories of < $15,000, $15,000 - $19,999, $20,000 - $24,999, $25,000 - $34,999, $35,000 - $49,999, and ≥ $50,000.

### Data management and analysis

Ideal for interpreting qualitative data in teams, we used the multi-step Framework Method for analysis [27] and applied a refined version of Kilbourne and colleagues’ health disparities research model [13].

First, each IDI and FGD audio file was transcribed verbatim into a Microsoft Word document. The FGD done in Spanish was translated into English by the bilingual interviewer who moderated the discussion in Kern County. It was then read and quality checked by our Spanish-speaking research assistant with native and advanced proficiency in English and Spanish. For translations of the most meaningful language parts in the findings, such as narrative about cultural health belief systems, and to reach resolution on questions related to translation accuracy, we used a back and forth communication process involving the interviewer (who moderated the FGD) and the native speaking researcher reviewing the transcript. Through ongoing discussion, the interviewer was asked to explain (to the quality-checking research assistant) the intended meaning of unclear words and passage translations, and provide detail on their context in the source language of Spanish. To arrive at the most correct and meaningful translations, possible wordings were discussed. Further, when use of fixed, one or two word translations (from Spanish to English) created challenges or altered meaning of what participants expressed, we instead described the content of what was narrated (in Spanish), using various English formulations to reduce the loss of meaning in the findings. Similar steps for maximizing representation and understanding, and enhancing validity of qualitative research conducted in Spanish and translated into English have been recommended by others in the field [28].

All transcript files were imported into QSR NVivo V12. Second, a few of each transcript type (i.e., IDI and FGD) were read by two team members to become familiar with the content and develop an overview of main ideas in the data. Two team members independently read the same two IDI and same two FGD transcripts and drafted coding labels, noted ideas and questions and highlighted segments of text referencing issues and content related to the research questions. These team members subsequently met to discuss the labels assigned to each passage, discrepancies between coders, refinements and/or additional codes required. Recurring ideas were collated into groups of similar themes and a final set of codes (with brief definitions) was agreed upon and organized into a conceptual index that was used to code remaining transcripts. New codes were recorded until no new codes were generated, resulting in a final analytical framework with 2 main categories, 2 sub-categories and 8 codes.

Category one (detection of disparities) was established to identify how participants understood and contextualized disparities surrounding risk for infection and congenital transmission of syphilis and had two codes: definition of health disparities and definition of vulnerable population. Category two (understanding disparities) was established to explore health system-level determinants of CS disparities at the patient-level, i.e., patient preferences (sub-category 1) and at the clinic-level, i.e., patient-provider encounters (sub-category 2). Sub-category 1 had 3 three codes: Feeling respected and empowered by the provider, feeling informed, and receiving care that reflects values. Sub-category 2 had 3 codes: patient-provider communication, interpersonal connection/emotional and interpersonal connection/professional.

We applied the analytical framework by indexing all transcripts, using the established categories and codes. An Excel spreadsheet was then used to generate a matrix for ‘charting’ the data by summarizing it (by category) from each transcript, allowing for interpretation.

## Results

We begin the results section with an overview of key characteristics about the participants. The remainder of findings are organized by the structure of the analytic research framework [13], starting with a description of participants’ definitions of health disparities and vulnerable populations in each setting. Next, we share results on perinatal patient preferences and their understandings of health system-level determinants of CS disparities. This section is organized by participants’ narratives about their feelings of being respected by providers and empowered to understand and make health choices; feeling informed about health and medical options; and receiving care that reflected their values of ethnicity, culture and family context. Last, we share findings on health system-level determinants of CS disparities at the clinic-level. Detailed narratives are provided on participants’ perspectives about clinical encounters in the context of prenatal care and maternal syphilis testing and treatment, including assessments of emotional and professional interpersonal connections between patients and providers.

### Participant characteristics

#### Prenatal care provider participants

Twenty IDIs were conducted with PCPs across both sites (10 IDIs per site). In Kern County we interviewed 1 prenatal care clinic supervisor, 1 public health medical investigator, 2 nurse practitioners, 3 public health nurses and 3 obstetricians/gynecologists (OB/GYN). In Baton Rouge we interviewed 1 maternal-fetal medicine specialist, 1 women’s health nurse practitioner and 8 OB/GYNs. Most providers were in a single-specialty practice (70%) and had been working for more than 5 years (60%). The majority of patients served by providers in our sample were Black or Hispanic (70%) and earned less than $20,000 per year (85%). Full details of IDI participant characteristics are shown in Table 1.

**Table 1:** Characteristics of In-Depth Interview Participants (Prenatal Care Providers) in Kern County, California and East Baton Rouge, Louisiana

	***Kern County (n = 10)***	***East Baton Rouge (n = 10)***	***Total (n = 20)***
**Type of practice**			
Single-specialty group practice	60% (6)	80% (8)	70% (14)
Community or public health clinic	40% (4)	20% (2)	30% (6)
**Years in practice**			
1 to 5 years	30% (3)	50% (5)	40% (8)
More than 5 years	70% (7)	50% (5)	60% (12)
**Race/ethnicity of most patients served**			
White or Caucasian	40% (4)	20% (2)	30% (6)
Black or African American	20% (2)	70% (7)	45% (9)
Hispanic/Latinx	40% (4)	10% (1)	25% (5)
**Income level of most patients served**			
Under $20,000	90% (9)	80% (8)	85% (17)
Between $20,000 and $49,999	10% (1)	10% (1)	10% (2)
$50,000 and higher	0% (0)	10% (1)	5% (1)
**Percent of patients uninsured or publicly insured**			
< 50%	0% (0)	40% (4)	20% (4)
50–99%	60% (6)	10% (1)	35% (7)
100%	40% (4)	50% (5)	45% (9)

#### Pregnant and postpartum participants

In total, we conducted eight FGDs, five in Kern County and three in East Baton Rouge Parish, with a total of 84 participants. All FGD participants in California and Louisiana self-identified as women and we refer to them as “women” and “participants” throughout the results section of the paper. Forty-two women in Kern County participated in focus groups, comprised of 5, 8, 9, 10, and 10 participants. Forty-two women in East Baton Rouge Parish participated in focus groups comprised of 12, 15, and 15 participants. At the time of interview, 54% of participants were pregnant and 46% had delivered in the past 12 months. The majority were Black, Hispanic or Indigenous (81%) and 90% reported an annual income of $20,000 or less. Full FGD participant characteristics are shown in Table 2.

**Table 2:** Characteristics of Focus Group Discussion Participants (Pregnant and Postpartum Women) in Kern County, California and East Baton Rouge, Louisiana

	***Kern County (n = 42)***	***East Baton Rouge (n = 42)***	***Total (n = 84)***
**Age**			
18–29	50% (21)	98% (41)	74% (62)
30–39	43% (18)	2% (1)	23% (19)
40–49	7% (3)	0% (0)	4% (3)
**Childbearing Status**			
Currently Pregnant	12% (5)	96% (40)	54% (45)
Delivered past 12 months	88% (37)	4% (2)	46% (39)
**Race/Ethnicity**			
White (non-Hispanic/Latinx)	36% (15)	0% (0)	18% (15)
Black or African American	12% (5)	96% (40)	54% (45)
Hispanic/Latinx	40% (17)	4% (2)	23% (19)
American Indian or Alaska Native	7% (3)	0% (0)	4% (3)
More than one race	5% (2)	0% (0)	2% (2)
**Annual Income**			
< $15,000	93% (39)	62% (26)	77% (65)
$15,000 - $19,999	2% (1)	24% (10)	13% (11)
$20,000 - $24,999	0% (0)	8% (3)	4% (3)
$25,000 - $34,999	0% (0)	2% (1)	1% (1)
$35,000 - $49,999	0% (0)	4% (2)	2% (2)
≥ $50,000	2% (1)	0% (0)	1% (1)
Not reported	2% (1)	0% (0)	1% (1)

### Defining health disparities

From a national perspective, providers from both locations knew certain areas of the U.S., including their own regions of operation were disproportionately and negatively impacted by STIs, including primary and secondary infection and congenital transmission of syphilis.

*It's a known fact that here in Kern County there is a much higher rate of sexually transmitted diseases - especially in teenagers. And even though in other parts of the country syphilis has are already been completely eradicated, in Kern County it's still an epidemic. It is like it (syphilis) is endemic because it cannot be completely cleared up. –* PCP in Kern County

Closer to home, providers and perinatal women in both sites conceptualized health disparities in terms of differences related to geographic layout in their own jurisdiction. Participants associated distribution and allocation of health resources (e.g., the local public health system, availability of and proximity to health care services, reliable transportation to care) with membership in a high- or low-risk groups.

*The health department is supposed to have the manpower to go out for home visits, but [sighs] I think there have been a lot of cutbacks on resources. It’s a shame because I think it shouldn't be conceived as an expense to get people treated. –* Perinatal participant in Kern County

Providers and perinatal women in both settings felt disparities in risk for syphilis infection and CS were both caused and exacerbated by societal inequities, primarily SES/poverty.

*For the majority, but not all, patients with syphilis, poverty is usually the leading thing. Poverty leading to lack of education and a lack of ability to make adult choices and that you get into positions where you're doing things that are more dangerous for sexually transmitted diseases. –* PCP in Baton Rouge

### Defining vulnerable populations

The way participants characterized vulnerable populations confirmed that the CDC definition we used for recruitment was accurate, but incomplete. Specifically, participants agreed pregnant women were at high-risk for syphilis infection if they lived in a high-morbidity region and/or practiced risky sexual behaviors, used alcohol and/or other drugs, had been incarcerated or interacted with the criminal justice system and/or had a history of infection with syphilis or other sexually transmitted disease (STD):

*Women who are positive for other STDs - like gonorrhea, chlamydia (are at increased risk for syphilis). If they’re getting these other STDs they're also at risk for syphilis. –* PCP in Baton Rouge

Persons were labeled as ‘high-risk’ if they had unprotected sex (i.e., without a condom), multiple sexual partners, sex partners who had multiple *other* sex partners, and/or if they had a sex partner currently or previously living with syphilis or another STI/STD.

*“High-risk” women are … definitely women with more sexual partners or who have unprotected intercourse or other high-risk behaviors that may result in either of these things (more sex partners and unprotected sex) being increased. –* PCP in Baton Rouge

Alcohol and other substance use were repeatedly mentioned as precursors to and consequences of sexual and reproductive health problems, including STIs. In Louisiana, providers felt marijuana was the most commonly used drug among their pregnant patients.

*Marijuana is on the rise now because it's been legalized in so many states. People think it's safe and it's being used pretty commonly as an antiemetic in the first trimester. So we acknowledge that about them [the patients] and they smoke some marijuana. It helps them with their nausea. –* PCP in Baton Rouge

In Kern County, methamphetamine was mentioned as the most significant problem among substance using pregnant women. Providers expressed concern about its negative impact on the health of the user and her pregnancy and how the drug fostered hyper-sexuality and aggression in relationships.

*The hyper-sexuality of meth use has increased STDs in Kern County because there is a correlation between high methamphetamine abuse and STDs. I mean not to say that other drugs don't contribute, but when you have a drug that the side effect is a stimulant, you know, you're not--if you're high you're not going to think of protecting yourself … if you're in a domestic violence relationship where both people are abusing (methamphetamines). Are they using protected sex? Are they cheating on each other? Are they telling each other that they're not, and then who has what? –* PCP in Kern County

Expanding on the CDC definition of vulnerable populations, four other subgroups were highlighted as warranting focused syphilis prevention efforts. These were racial/ethnic minority populations, people with transient vulnerability due to currently (but potentially time varying) limited economic or social resources (e.g., immigrants and people experiencing homelessness), people with a history of other (i.e., non-STD/STI) health conditions, and youth.

Both providers and perinatal participants explicitly referenced racial and ethnic minority status when defining vulnerable populations. Some FGD participants felt this was the single biggest challenge to easily accessing high quality prenatal care.

*Being Hispanic (is the biggest problem pregnant women face when trying to access prenatal care). I feel like the people around who are Hispanic - and you know, those who came in illegally - they may all eventually get health care. It's just that you have to put in more work/more paperwork. –* Perinatal participant in Baton Rouge

Also included in providers’ definitions of vulnerable groups (and related to SES, but not necessarily associated with historical experiences of disparity) were immigrants, refugees, and people experiencing homelessness. One OB/GYN from Kern County felt fear of deportation prevented many undocumented pregnant women from receiving adequate prenatal.

*There are undocumented people who work on the farms. The migrating farm workers. [It would reduce their risk for] somebody to go out there and offer cheap care … . just have a screening. If someone comes up positive, we're not going to report you, it's going to be confidential, and you get free treatment. That's my thought about what could be done for these poor people who are afraid to come to the clinic. –* PCP in Kern County

Unstable housing, whether temporary or more long term, created a substantial gap in pregnant women’s use of and retention in prenatal care.

*I was homeless. When I found out I was pregnant, I didn’t go in right away to see an OB/GYN. It actually happened, like, when I was 5 months. And then after that, I started going to see the OB/GYN. But I didn’t do very well with going to appointments. They made appointments; I just didn’t follow through all the time. I got my ultrasound and all that stuff. But I was homeless, so it was like, I missed my appointments half the time. Even though I was right by it (the health center), I didn’t make it to all my appointments. Being homeless made it more difficult. –* Perinatal participant in Kern County

Vulnerable populations were also described according to a range of non-STI health needs contributing to disparities, such as having an existing health condition like diabetes, high blood pressure, or obesity.

*We see patients with a myriad of problems which can include a sexually transmitted disease while, at the same time, they have diabetes and hypertension. It's hard to prioritize when any one of those three could cause major health problems, either long term or acutely, both for the mother or for the child. –* PCP in Baton Rouge

Lastly, youth were flagged as a key population often missed by providers when it came to screening for syphilis - presumably inadvertently, due to assumptions that the young person would not be pregnant.

*The population we're missing often is young adults. They come in with signs of STIs but sometimes the physicians are only ordering PCR tests for gonorrhea and chlamydia and missing the syphilis testing … . Last week we had a young mom who was positive. She was less than 21 weeks pregnant and had missed the syphilis testing. There is opportunity for education with our outpatient providers who are seeing mothers of all ages potentially coming in. –* PCP in Kern County

### Perinatal patient preferences for health and prenatal care

Provision of care that respects and responds to patient preferences is at the core of a patient-centered approach [12]. The influences most commonly mentioned by women as impacting their feelings about and choices related to prenatal care were: (1) Feeling respected by the provider and empowered to understand and make choices about their health; (2) Feeling informed about their health and medical options; and (3) Receiving care that reflected their values of ethnicity, culture and family context.

#### Feeling respected and empowered by the provider

Feeling respected by a provider was identified as a core element of quality prenatal care and something that facilitated use of and adherence to all health services. Women felt respected by providers who listened to them, did not pass judgment, made them feel cared for and who offered a safe space to ask questions and discuss concerns without being rushed or patronized.

*My nurses are real nice. They call my doctors there the same way, like, we’re sitting here having a normal conversation. It’s not all the medical terminology and all that. She actually gets to know who you talk to. The nurses come in like, after the first time, they remember your face and they greet you, like, “Oh hey, Monica, how you doin’?” Like, it’s really—I have no problem talking to them about ‘this is happening and that is happening,’ You know, it’s like, I don’t feel like I’m being low-key judged or like, looked at funny, you know? –* Perinatal participant in Baton Rouge

Self-respect was also mentioned as an important factor in seeking and receiving high quality care and making medical decisions, such as those surrounding STI testing and treatment, especially during pregnancy. This is illustrated in the passage below, by a woman from Louisiana who explained how feelings of self-worth and confidence were essential for both taking care of yourself and for others.

*You might be afraid of the way someone will look at you or the way someone will feel about you and say “I don’t feel like (going to the clinic for care) … ” But no woman should ever be afraid of getting help or seeking help or medical attention for something that you have … for something that you’re dealing with - physically, in your body. Especially ‘cause, you know it’s not just you now. It’s also someone that’s on the inside of you that you are worried about. –* Perinatal participant in Baton Rouge

#### Feeling informed about health and medical options

In both California and Louisiana, women demonstrated limited knowledge about STIs. For instance, most remembered basic facts about sexual and reproductive health (i.e., that using a condom could prevent pregnancy and transmission of an STI) but few could distinguish between syphilis and other STIs. However, women in both sites were overwhelmingly aware of syphilis, that it was an STI, and that it was linked with adverse health outcomes. Fewer women knew the specifics of those adverse health outcomes, that the infection could be transmitted from a pregnant woman to her baby or what impact it could have (on the baby). There was also general confusion about when syphilis and other STI testing should be done during pregnancy.

*I have had education on syphilis and stuff in the past, but not what it can do to the baby. I know if you’re not pregnant, it can make you sterile or you won’t be pregnant because of the medications they give you. As far as pregnancy, no doctors have discussed pretty much any STD with me, or tests, and when you’ll take the next one, and so on. –* Perinatal participant in Baton Rouge

Irrespective of knowledge levels about STIs and syphilis, perinatal participants explicitly wanted to be informed of and involved in decision-making surrounding the health of themselves and their babies. Women in both settings preferred being informed through face-to-face interaction with their providers, relative to phone conversations or learning from written materials. Participants felt in-person provision of information increased the likelihood that a patient would be exposed to health content, relative to a phone call (where you cannot pick up on body language) or handing out brochures to read. An FGD participant in Kern County preferred face-to-face communication with her provider (versus learning from written materials) because *“It seems more personal. If you are interested in the problem, you will read it, if you are not interested, you will throw it away.”* However, most women reported having limited time with their providers - due to infrequent visits and/or short in-person clinic sessions. Thus, pregnant patients were commonly left to rely on brochures, pamphlets and other written materials. While convenient, these materials were commonly found to be confusing and overly technical and most women preferred having providers explain their contents or offered time to discuss and answer questions.

*I received all that information and they kept giving me additional information. But, I’m more hands-on, so you have to talk to me after I read something, So when you do the first blood (draw), you can’t just stick me up a needle. They had to tell me what each one was, but they didn’t give me a pamphlet on it, I needed them to talk about it. My doctor did explain … the reason for these tests, and when you’ll take the next one, and so on. –* Perinatal participant in Baton Rouge

Differences in literacy and English fluency created additional, related gaps in health knowledge, particularly among individuals whose providers relied on written materials (and/or only provided materials in English) to educate their patients. This was noted to have substantial impact on the large number of Spanish-speaking people in Kern County who had limited (or no) English skills. The topic came up in a discussion about challenges perinatal women faced when trying to find prenatal (as well as pediatric) health care providers and understand the information they provided.

*FGD participant 7: The majority of pediatricians I looked for did not speak my language. Then it was difficult to understand the doctor or my baby's pediatrician.*

*FGD participant 3: The problem I see - and a lot of people too have told me this, it’s not just me - is the language. Sometimes they’ll give you information but it’s in English, so - how are you going to understand it? You ask them “is there a translation?” They said, “no, we only have the information in English.” Yes, it is a problem.*

Gaps in literacy also created health care access disparities for people with low levels of education, including - as noted by a perinatal participant in California - *“a lot of homeless people who try to go in for medical coverage but can’t read or write.”*

Most women also sought information from the Internet. Almost all perinatal participants looked for facts on Google and/or other search engines. Many downloaded pregnancy apps on their phones, to learn about prenatal nutrition, fetal development, breastfeeding tips, and pregnancy and newborn milestones.

*The Baby Center app, that’s a really good app, and the Pregnancy Plus app. I would really recommend it. It goes with you, week by week. It shows you the baby’s developments, shows you things you might be going through, things that will help. You know, a lot of questions that we might have to answer. Because on Baby Sitter, it has a search engine like you would search Google, but it’s all baby information that you could really get help with so I highly recommend that app. –* Perinatal participant in Baton Rouge

Online “research” was described as convenient (i.e., it can be done at any time from any location) and to feel safe (i.e., it did not involve potentially uncomfortable interaction with a provider). Further, some perinatal participants said it was their preferred mode of education and information because they did not fully trust what they were being told and advised to do by their prenatal clinician(s).

*I like going to my doctor sometimes. I mean, I will only go to see what their input is on a situation, but I have found that - a lot of the time - they just kind of guess or they just, you know, say the first thing that comes to mind. I've had a couple of bad experiences where I had to go out and do my own research and come to my conclusions. And there's several instances where I did reach out to the health providers and I was misled. –* Perinatal participant in Kern County

The WIC Program was discussed in all focus groups (in both states) as not only a key supplier of food to low-income women and their families, but also a common source of accessible and judgment-free education on nutrition, pregnancy and childbirth, and breastfeeding and as link to social services referrals.

*You receive a lot of help from WIC because they refer you to class or something like that … to help you out and better your life of being a parent. WIC helps a lot with being a parent and healthy things, the way you eat, what your baby eats. –* Perinatal participant in Baton Rouge

#### Receiving care that reflects values of ethnicity, culture and family context

Participants highlighted the importance of providers recognizing that some patients have cultural health belief systems that differ from their own. They were not advocating for PCPs to provide differential treatment, but to understand how cultural issues influence patient understanding of what causes illness, how illnesses are cured, and who (e.g., family, medical provider) should be involved in the process of diagnosis and treatment. Cultural health belief systems influence choices surrounding use of care and compliance with medical recommendations. Several Hispanic perinatal participants, for example, explained how they were reluctant to share information about their sexual and reproductive health with their PCP because they were brought up to learn it was inappropriate to discuss intimate matters with people outside their marital/intimate relationship or their family.

*In my culture and my home I was educated to not talk about things like sexuality and menstruation or other things … how do you say it … other things about a woman’s reproductive health. Because of this, it has been difficult for me to talk with my doctors about these kinds of things. I struggle to open up, especially with a male doctor. Sometimes you have a male doctor and other times a female doctor. Like the other day there were more female than male doctors in the clinic. So I talked to a female doctor and it was much easier for me to open up because I felt I could tell her exactly what was going on, but I can’t tell everything to a man. My mother told me it was not good to tell certain things to men. –* Perinatal participant in Kern County

Spiritual and religious influences, and beliefs in traditional folk illness, played a role in some women’s comprehension of health and illness. FGD participants in Kern County, for instance, narrated how sickness was often associated with folklore and/or spiritual elements in Hispanic cultures. One woman explained how this system extends beyond STIs to influence a range of health beliefs, from common colds to chronic health problems. Using cancer as an example, she said her family members were convinced cancer is contagious, stating, “*Because of our culture, they think in a superstitious way and believe the disease could be passed from one person to another.”*

Beliefs about causes of illness influenced a patient’s choice of provider and treatment and women wished this was respected by more clinicians. Participants shared how seeing a provider who lacked cultural awareness influenced some pregnant women to avoid using services (due to fear of being misunderstood or disrespected) and/or ignore medical advice (due to not understanding or trusting the provider).

*I've had, like, way too many bad experiences like this with doctors. I would rather not see a doctor at all. –* Perinatal participant in Kern County

Prenatal providers also recognized false/misleading information as a barrier to CS prevention among vulnerable pregnant women, particularly women with unequal access to health care information. An OB/GYN from East Baton Rouge explained how a large number of her patients got most of their pregnancy health information from unreliable sources, like medical folk wisdom and social media.

*I’ve never spoken to a patient that has gone to an actual educational website, that I would refer them to, and sought out information about x,y,z. There are a lot of old wives’ tales, I think. Like getting Trichomonas from a toilet seat. We hear that all the time. Maybe not in so many words, but - yes - all the time. And I don't think someone read that. It’s more like ‘my friend told me this or my aunt told me this.’ –* PCP in Baton Rouge

Building on the above quote, family had a significant influence on some participants and it was important that their perspectives not be negated by providers’ recommendations. For instance, and as illustrated above, older female relatives (e.g., aunts, mothers, grandmothers) often served as a main source of trusted knowledge and guidance on important matters related to intimate and sexual relationships, as highlighted in the FGD dialogue (from Baton Rouge) about respecting mothers’ and grandmothers’ pregnancy advice.

*FGD participant 1: I don’t go against Grandma’s words.*

*FGD participant 2: She’s always right.*

*FGD participant 1: Grandma’s the one who’s going to say (to you when you are pregnant), “Oh girl, don’t reach over your head. Don’t you do this. Don’t you … ”—you know.*

*FGD participant 3: I agree with them. When you don’t know … your mothers know.*

### Perceptions of patient-provider clinical encounters in prenatal care

Perinatal participants expressed their feelings on how their health preferences were responded to by their providers and the health care system. Both providers and perinatal participants shared perspectives on exposure to other elements of their health-care relationships, including patient-provider communication, and what has been described elsewhere as emotional and professional interpersonal connection [29] during prenatal care.

#### Patient-provider communication

All participants felt respectful communication was critical for successful patient-provider relationships and pregnant women’s acceptance of and adherence to care. This said, providers frequently struggled to discuss difficult or complex topics with high-risk, low-income patients, without running short on time allotted for the visit and/or becoming impatient/agitated. A few FGD participants said their prenatal provider made them feel very uncomfortable by displaying a sense of uneasiness in their presence.

*I started off with a male doctor. Kinda older. And … I don’t know, I just felt SO, SO, SO uncomfortable. … .by certain things he would respond to … or how he would act as if HE was nervous being around me. That’s what made me find out about a midwife and now, always when I go to the doctor, my midwife is also there to help me feel comfortable around certain doctors. I prefer women doctors. -* Perinatal participant in Baton Rouge

To compensate for these shortcomings, providers in both states often relied on external services, run independently or through the local health department, to provide enhanced prenatal care. For example, providers in Kern County often liaised with the Comprehensive Perinatal Services Program (CPSP) which offers free services (e.g., nutrition education, prenatal vitamins) to pregnant women eligible for Medi-Cal.

*The CPSP people are specially trained and have time to do deal with social issues for the patient: How to get the medicine. How to arrange their Medi-Cal. How to do the paperwork. You know, it's all kind of personal … . As a provider, we are more official. These people are just...rather, kind of like, lay people. They use lay people language and are not perceived by the patient as the official. It's a good interface between the health care system and the patient population. And, you know, they call them, they are bilingual. They share the same cultural background and the communication is more patient-friendly. –* PCP in Kern County

As reflected in the quote above, providers recognized the importance of plain (i.e., “lay people”) language for enhancing health literacy. Despite this, some participants felt they were given incomplete information by their providers, or they simply struggled to understand what their prenatal care provider shared about their (or their children’s) diagnoses, test results, suggested treatment, etc.

*Because all they tell me are the negative consequences. That’s why I can’t see what it is that I am giving to my son. –* Perinatal participant in Kern County

Further, a few women said their provider “talked over” them, used confusing terminology or medical jargon and made little or no attempt to empower them to take control of health care choices for themselves or their children. Women preferred providers who helped them increase their own health literacy by communicating with them (using plain language in both written and verbal communication) about how to obtain, process, and understand basic information about their health and available services, enabling them to make informed decisions.

*Many times when my child gets sick the doctor will talk to me but I do not understand what he is saying. I do not understand the medical language being used. For me, it would be more useful if he explained the problem and what the consequences are or what benefits are possible with medicine. –* Perinatal participant in Kern County

#### Interpersonal connection

*Emotional interpersonal connections* were described by perinatal participants as bonds created with providers who took time to get to know each patient and treat them as an individual. A positive example was offered by a woman from Kern County who had been seeing the same doctor for 8 years. She shared how she had recently brought her friend into the clinic to become a new patient and (while there) ran into her doctor who *“knew me by name”* and inquired *“How are you doing?”* This made her feel even more connected to her provider and fully confident she would offer excellent care to her friend.

While women from both sites indicated they wanted clinicians to demonstrate compassion and empathy towards them, most said they frequently had negative experiences with their prenatal care providers, as well as administrative staff in the health system. Perinatal participants in our study knew they were characterized as “high-risk” for CS and overwhelmingly felt overlooked by providers and the system. Instead of being offered services where emotional interpersonal connections were established, women said they frequently received little or no continuity of care in terms of having a regular clinic to visit and/or seeing the same provider each time.

*When I was at [name of clinic] I did not know who my doctor was. Every time I went, I saw a different one. Whoever got me would say “let’s see if you will see the same doctor next time,” but I would never see the same doctor the next time. It was always a different one and some doctors would go very fast, even when I had more questions. They would hurry you and I felt pressure so I would say “no, thanks” (I don’t have any questions) because they did not give me enough time. –* Perinatal participant in Kern County

Such discontinuities in prenatal care reduced women’s sense of being valued by their provider and contributed to a sense of being unworthy of the provider’s time.

*It's a matter of the relationship and the bond you build with your doctor, because with some doctors, you don't really feel comfortable. It's like they're trying to get you in and get you out. Like they don't have the time to sit there and actually engage in conversation because it's about the next customer or the next patient or the money. –* Perinatal participant in Baton Rouge

*Professional interpersonal connections* were referenced as bonds of trust whereby providers believe patients will be truthful and forthcoming with health information, and patients believe providers will do everything to uphold their best interests, irrespective of any vulnerabilities they may have.

Most providers expressed deep commitment to offering care that prioritizes patients’ needs, preferences, and values. Female providers were most likely to share how they strived to meet prenatal clients ‘where they are’ and take time to understand each pregnant woman’s medical history, current situation, and any factors influencing their navigation of the healthcare system – without judgment. A provider from Kern County Public Health Services Department referred to her office as *“a no judging zone,”* stating “*You come here because you need something and we’re here to fulfill that need. We’re not here to judge you.”* Despite this, a few participants felt that persistent shame was placed on some vulnerable groups which adversely impacted their use of prenatal care.

*I was homeless during my last pregnancy and, if somebody is homeless, people tend to look at them like they're dirty. I mean they* ***are*** *dirty but, still, people treat them like - you know - like they're pretty much beneath us. And so, homeless people tend to not want to try and go get help. –* Perinatal participant in Kern County

The value of transforming the medical community to be more supportive of people who are frequently marginalized, such as people with substance use disorders or people experiencing homeless, was recognized as both complicated and necessary.

*When you're working with homeless women or drug-using moms, it can be delicate to engage them because there's so much shame around it. You'll see these moms who will wait until they're 40 weeks pregnant to go in for care, with zero prenatal care before that. It is because of the shame. –* PNP in Kern County

An OB/GYN resident in Louisiana said her team aimed to serve as a bridge between patients from the community and their institution’s providers. To build professional interpersonal bonds with patients, they appointed a local women’s health advocate to help the clinic establish relationships and create trust with local women. Goals were to open up dialogue between local women and the institution and break down any barriers that might prevent pregnant women from seeking prenatal care.

*Our goal was to say “look, come here to [clinic name]. You know this is the place where we live and work. This is why we are here, and you know – we are doing this for you. How can we do it better?” That was literally the theme of it – ‘how can we do this better?’ The woman that came (the women’s health advocate) was incredible! She was awesome and she had so many insights. She was very motivated also to help women in her community. –* PCP in Baton Rouge

Overwhelmingly, providers felt they were doing their best to offer attentive, open-minded care to all patients. At the same time, a few of their narratives suggested unrecognized biases against patients within some of the most marginalized groups, namely women using drugs and/or women involved in transactional sex. These potentially implicit biases seemed to influence providers’ trust in their patients’ accounts of their own behavior and ability to recount their own histories. For instance, when asked how he handles patients who might be using drugs, an OB/GYN in East Baton Rouge responded:

*If we have a positive drug screen for meth - which so often they deny - we have to get a thorough history. If we can believe their history, we’ll refer them to our social services.*

This same provider also lacked confidence in the veracity of his patients’ responses to his inquiries about their potential involvement in sex work.

*First of all, it is hard to get them to admit it, but there are certain little hints and signs that may be involved. But we get our social services people involved.*

Although such biases may operate in an unintentional, even unconscious manner, and may be grounded in accuracy (i.e., it may be true that substance users deny using drugs), such stereotyping can lead to clinical decision-making that negatively impacts patient care and/or perpetuates patient mistrust in the provider.

## Discussion

This study engaged prenatal care providers and perinatal women from two of the highest congenital syphilis morbidity regions in the U.S. Guided by a health services analytic framework [13] our goals were to assess how participants understood the concepts of ‘health disparities’ and ‘vulnerable populations’ – as they related to their own, lived experiences with providing and receiving maternal and perinatal health care (including syphilis testing and treatment) - in high-risk geographic areas. We also sought to examine health preferences of perinatal women in our sample and garner their perceptions about their relationships with prenatal providers and quality of clinical encounters. Four main findings emerged. First, some determinants of CS disparities were common to both populations, while others were specific to Kern County or East Baton Rouge Parish. Second, although perinatal women in both sites had clear health preferences, including patient-centeredness, receipt of care that respected and responded to these preferences was inconsistent. Third, some of these inconsistencies were connected with ethnic and cultural traditions and others were linked with seemingly implicit, negative attitudes toward patients who were using substances, experiencing homelessness, or engaging in sex work. Fourth, while providers aimed to offer high quality care to their patients, numerous issues precluded most perinatal participants from having consistently positive, communicative relationships with their providers which contributed to gaps in the cascade of prenatal care and STI testing and treatment.

Participants agreed with the CDC’s widely accepted definition of pregnant women being at highest risk for syphilis if they had a history of past (syphilis) infection, incarceration, drug use, or multiple or concurrent sex partners [23]. Research from several other studies in the U.S. [30], including California [31] also identified some or all of these same factors as determinants of risk for syphilis in pregnant people. In our study, we learned that the definition of vulnerable population, as related to CS risk in Kern County and Baton Rouge, was incomplete without also including racial/ethnic minority populations, people experiencing transient economic and/or social resource limitations, people with a history of other health conditions, and youth. These findings build on a small body of research, done in the U.S. and published over the past decade, suggesting African-American/Black ethnicity [32, 33], Hispanic ethnicity [34], and homelessness [35] increase risk for CS. Large-scale health disparities research is warranted, to generate national population-level estimates of the scope of CS, setting-specific determinants and to assess differences in race, ethnic background, income-level and socioeconomic status, drug use status, education level, disability status, etc.

A few site-specific distinctions emerged in our findings on definitions of vulnerable population. For instance, while a linkage between CS and substance use was reported in both locations, the main drugs correlated with STI risk differed by state. In California, methamphetamine was most commonly associated with syphilis transmission while in Louisiana it was marijuana. Two noteworthy points are that marijuana and methamphetamine use were also connected with CS in California and Louisiana (respectively) but providers, in particular, prioritized their concern with one drug over the other. Second, in January 2018 – 4 months before we launched our study - legislation was passed in California allowing people to legally purchase marijuana and cannabis products, which may have influenced these trends. Looking at the 5 years preceding our study, national data from 2013 to 2017 found a substantial percentage of syphilis transmission in the U.S. occurred in people who used methamphetamine, injected drugs or had a sex partner who injected drugs or used heroin [36].

Another site-specific finding related to the majority Hispanic/Latinx population in Kern County and large number of people whose first language was Spanish. Reduced health knowledge was linked with language barriers and limited availability of providers and educational materials in languages other than English. This also contributed to disparities in perinatal participant’s ability to communicate with their providers and be fully involved in their health care and medical decision-making. Two important take-home lessons emerge here. One is that to truly offer high quality patient-centered care, it is essential that language barriers be addressed, either through provision of medical interpretation or availability of bi/multi-lingual providers and educational materials (or both). Two, to reduce CS disparities, evidence-informed interventions should be customized to address the setting-specific characteristics (including low literacy and substance use) that preclude pregnant people from getting prenatal care, STI testing and treatment.

To understand the origins of the disparities we saw in prenatal care use and risk for syphilis infection in Kern County and East Baton Rouge Parish, we (previously) explored and reported on potential socioecological and structural explanations. These included differences in SES and ability to pay for out of-pocket health care costs, access to reliable and affordable transportation, access to healthcare insurance, sexual health literacy and gender equality [6, 7]. It has been posited that patient preferences may also explain some disparities in health care use [37] and we, indeed, found perinatal patient’s health beliefs were related to their decisions about maternal health, prenatal care and STI testing and treatment. Similar to results from the notably distinct context of urban Canada [38], we found health preferences and perceived emotional and professional connections with a provider to have as much or more impact on perceived quality of prenatal care as structural issues (e.g., transportation and health insurance coverage).

Although perinatal participants in both settings had low levels of STI knowledge, all had well-formed opinions about what was important to them related to choices about their health and medical treatment. Women in our study wanted providers who respected them and empowered and encouraged their active involvement in their own health care planning and decision-making. It was important to women to feel informed about their health and medical options, and to receive care that reflected their health preferences, personal and family values, cultural traditions, and socioeconomic conditions. However, apart from a few participants who expressed happiness with the health services received during their most recent pregnancy, most felt they were provided low quality care. Most women reported late onset of prenatal care (i.e., after the first trimester), limited in-person interaction with their providers, missed appointments and/or gaps and delays in STI screening and/or treatment. Our findings agree with national data that suggests the two most commonly missed opportunities for prevention of CS are a lack of adequate maternal treatment despite timely diagnoses of syphilis and a lack of timely prenatal care [9].

Providers in our study unequivocally recognized the severity of the CS epidemics in their regions and expressed commitment to mitigating its impact on the vulnerable populations they served. Most had been in practice multiple years and were aware of the CDC and American College of Obstetricians and Gynecologists guidelines on screening, testing, and treating congenital syphilis [6, 7]. Nonetheless, many gaps remain in pregnant people’s use of and adherence to prenatal care in Kern County and East Baton Rouge Parish, not unlike what has been reported throughout the U.S. [9]. Echoing the conclusions of colleagues who explored women’s and providers’ perspectives of quality prenatal care in Canada almost 10 years ago, we feel our findings highlight the importance of not only focusing on structural and biomedical aspects of quality of care, but the need to also improve the quality of the patient-provider relationships, as well as the emotional and professional interpersonal connections [38]. Women in our study wanted providers who fostered a health care climate that enabled them, as patients, to control their own situations, manage their own care, and trust in themselves and their decisions. Thus, in addition to clinical training on CS prevention protocols, we recommend providers be supported to develop skills to enhance patient engagement, knowledge of the importance of patient autonomy and agency and skills to provide care that responds to patient preferences.

We also advocate for the training of providers on cultural diversity, competence, and awareness and the development of cross-cultural empathy. Our findings suggest that links between ethnic/cultural traditions and health preferences may contribute to disparities in use of and retention in prenatal care, including patterns of syphilis testing and treatment. For example, perinatal Hispanic women narrated a strong, shared cultural heritage that highly valued the roles of family, religion and spirituality. These characteristics influenced all aspects of their lives, including health behaviors. Hispanic women seeking prenatal care had typically already received advice from family members about what to do during pregnancy. Some women had learned (from respected relatives) it is inappropriate to disclose intimate information with people outside the family. Thus, some women withheld critical sexual and reproductive health details from their medical providers. Building stronger patient-provider relationships could establish/improve trust in the clinical setting and enhance women’s confidence in their prenatal providers. This in turn could enable clinicians to better understand patients’ cultural and family values and how they influence their health beliefs and preferences. Clarifying cross-cultural misunderstandings could also help providers make sure they order the right tests or offer needed medical advice/attention.

Inconsistencies in participants’ perceived quality of prenatal care were also connected with seemingly implicit, negative attitudes toward patients who were immigrants and/or were using substances, experiencing homelessness, or engaging in sex work. A great deal of social stigma and shame was attached with health care-seeking by pregnant women in all of these vulnerable categories. Although the providers we interviewed believed they were doing their best to be unbiased and non-judgmental, it is likely they – like people in the general population – hold unconscious stereotypes and attitudes toward certain groups of people. Research has found that these implicit biases can negatively affect patient-provider relationships and the decisions that clinicians make when making medical decisions and administering care and treatment [39]. Social vulnerabilities during pregnancy have been highlighted as particularly complex, with regard to how provider attitudes impact their relationships with patients, as well as their patients’ subsequent health behaviors and choices. Substance-using mothers, for example, have disclosed feeling less trust in providers with whom they’ve had negative experiences and they are less likely to comply with their care recommendations [40]. Encouragingly, research has found people, including providers, can address and change these unconscious biases by becoming aware of them in the first place, and then actively working to dismiss the biases that affect interactions with patients [39]. A growing number of implicit bias training programs are available for health care providers in all specialties. Additionally, clinical experts in the fields of high risk prenatal care, labor and delivery and neonatal intervention have published focused guidance to help clinicians provide competent and compassionate care to pregnant and postpartum women [41].

Our study has limitations. The sample size was small and restricted to two high morbidity CS regions in the western and southern parts of the U.S. Perceptions of perinatal women and prenatal care providers from other regions of the country, as well as other regions of California and Louisiana, would offer additional insight. Research to understand setting-specific dynamics is warranted. Another limitation is that our assessed definitions of CS health disparities and vulnerable populations were qualitative in nature and only based on input from a small number of people per setting. Thus, it is possible that differences exist between participants’ perceptions of risk factors (for acquiring and transmitting syphilis in pregnant women) and population-level estimates of evidenced risk factors. Unfortunately, data are unavailable on some of these site-specific statistics, such as CS rates among homeless women and/or CS rates among methamphetamine-using women in Kern County and East Baton Rouge Parish. Next steps in our research plans include exploring these important determinants. Another shortcoming of our research is we did not discuss or assess participants’ gender identity or sexual orientation. This gap precludes our ability to identify specific health needs of sexual and gender minority (SGM) patients in our study, and understand the health disparities they experienced. It also prevents us from understanding how gender identity and sexual orientation influences the patient-provider relationship, both in terms of how SGM patients receive care and how SGM providers serve their patients. Our future work will collect information on gender identity and sexual orientation, as we believe these data are essential for understanding what is needed for high-quality, patient-centered care. Lastly, we did not collect information on how providers identified their own race or ethnicity and are therefore unable to explore how these characteristics might have influenced their relationships and interactions with their patients. We will collect these details in all future research and recommend that other studies do the same.

## Conclusions

Congenital syphilis is a persistent public health and health disparities issue in the United States. A growing evidence-base has helped researchers and clinicians identify and address multiple social and structural determinants of syphilis infection in pregnant people. It is also important to understand setting-specific factors that protect or put pregnant individuals at risk for acquiring or transmitting syphilis so interventions can be tailored to meet the unique needs of each population. Additionally, attending to key health systems level dimensions of clinical care could improve high-risk pregnant people’s uptake of and retention in use of perinatal services. Focused, site-specific interventions are warranted to improve pregnant people’s access to prenatal care and ensure they and their sex partners receive timely syphilis/STI screening and all individuals who test positive receive immediate treatment. All prenatal care providers should receive comprehensive (refresher, if needed) training on procedures for diagnosing and managing congenital syphilis, identifying and mitigating implicit biases and on how to provide competent and compassionate patient-centered care.

## Data Availability

The datasets generated and analyzed in the current study are not publicly available to protect participant confidentiality but are available from the corresponding author upon reasonable request and with permission from the investigators and approving institutional review boards.

## Abbreviations

CS: Congenital syphilis
FGD: Focus group discussion
IDI: In-depth interview
OB/GYN: Obstetricians/gynecologists
PCP: Prenatal care provider
STD: Sexually transmitted disease
STI: Sexually transmitted infection
WIC: Women’s, Infants, and Children nutritional program

## Footnotes

1 We hand calculated this incidence rate, using figures from the Johnson, 2019 report [22]. Three different case numbers (43, 44 and 46) were presented throughout the report. We used the middle number (44) for our calculations.
